# Identification of cotton pest and disease based on CFNet- VoV-GCSP -LSKNet-YOLOv8s: a new era of precision agriculture

**DOI:** 10.3389/fpls.2024.1348402

**Published:** 2024-02-20

**Authors:** Rujia Li, Yiting He, Yadong Li, Weibo Qin, Arzlan Abbas, Rongbiao Ji, Shuang Li, Yehui Wu, Xiaohai Sun, Jianping Yang

**Affiliations:** ^1^School of Big Data, Yunnan Agricultural University, Kunming, China; ^2^College of Plant Protection, Jilin Agricultural University, Changchun, China; ^3^Jilin Haicheng Technology Co., Ltd., Changchun, China

**Keywords:** artificial intelligence, cotton, pests and diseases, deep learning, machine learning, XIoU, YOLO

## Abstract

**Introduction:**

The study addresses challenges in detecting cotton leaf pests and diseases under natural conditions. Traditional methods face difficulties in this context, highlighting the need for improved identification techniques.

**Methods:**

The proposed method involves a new model named CFNet-VoV-GCSP-LSKNet-YOLOv8s. This model is an enhancement of YOLOv8s and includes several key modifications: (1) CFNet Module. Replaces all C2F modules in the backbone network to improve multi-scale object feature fusion. (2) VoV-GCSP Module. Replaces C2F modules in the YOLOv8s head, balancing model accuracy with reduced computational load. (3) LSKNet Attention Mechanism. Integrated into the small object layers of both the backbone and head to enhance detection of small objects. (4) XIoU Loss Function. Introduced to improve the model's convergence performance.

**Results:**

The proposed method achieves high performance metrics: Precision (P), 89.9%. Recall Rate (R), 90.7%. Mean Average Precision (mAP@0.5), 93.7%. The model has a memory footprint of 23.3MB and a detection time of 8.01ms. When compared with other models like YOLO v5s, YOLOX, YOLO v7, Faster R-CNN, YOLOv8n, YOLOv7-tiny, CenterNet, EfficientDet, and YOLOv8s, it shows an average accuracy improvement ranging from 1.2% to 21.8%.

**Discussion:**

The study demonstrates that the CFNet-VoV-GCSP-LSKNet-YOLOv8s model can effectively identify cotton pests and diseases in complex environments. This method provides a valuable technical resource for the identification and control of cotton pests and diseases, indicating significant improvements over existing methods.

## 1 Introduction

Cotton is one of the vital fiber crops in China, extensively employed in textile production and the manufacturing of cotton goods. However, the cotton industry in China has been severely jeopardized by the pervasive threats of diseases and pests, leading to adverse impacts on the yield (Chohan et al., 2020; Abbas et al., 2021). Traditional methods of pest and disease detection often rely on seasoned experts who gauge the health of cotton leaves through visual inspection. Despite its widespread use, this conventional approach suffers from multiple shortcomings. First, these methods are labor-intensive and time-consuming, requiring significant human resources, especially in large-scale cotton cultivation. Second, the manual inspections depend on the subjective assessments of experts, introducing variability and compromising the consistency and accuracy of the results.

With the advent of advancements in computer vision technology and deep learning algorithms (Wang C. et al., 2023), the agricultural sector has witnessed new avenues for pest and disease detection (Meng et al., 2023; Ye et al., 2023). These technologies not only automate the identification process but also enhance the speed and accuracy of detections. Notably, the YOLO (You Only Look Once) algorithm (Jiang et al., 2022; Zhang Y. et al., 2023) has achieved remarkable success in this context, acclaimed for its real-time processing, multi-scale support, automation, and efficient data handling, thus providing a robust tool for pest and disease monitoring and management in agriculture.

Several researchers have made notable advancements in the field of cotton disease identification and monitoring. Caldeira, R. F. et al (Caldeira et al., 2021)used the convolutional neural network learning models GoogleNet and Resnet50 to monitor the health status of cotton crops, and obtained accuracy rates of 86.6% and 89.2% respectively.

Nannan Zhang et al. (Nannan et al., 2020) presented the CBAM-YOLO v7 algorithm, an improved attention mechanism YOLO v7, with a mAP of 85.5%, providing a strong theoretical foundation for real-time cotton leaf disease monitoring. Yuanjia Zhang et al. (2022a) developed a real-time, high-performance detection model based on an enhanced YOLOX algorithm. The comparative results also demonstrated that the improved model achieved mAP values 11.50%, 21.17%, 9.34%, 10.22%, and 8.33% higher than the other five algorithms, meeting real-time speed detection requirements. According to Liu and Wang (2020), the feature layer of the Yolo V3 model using an image pyramid to achieve multi-scale feature detection, resulting in improved accuracy and speed for the detection of diseases and pests in tomatoes. Zhenyang Xue et al. (2023) proposed YOLO-Tea, an enhanced model based on You Only Look Once version 5 (YOLOv5), outperforming YOLOv5s by 0.3% to 15.0% across all test data. Furthermore, Liu et al. (2023) introduced MRF-YOLO, a deep learning method with multi-receptive field extraction based on YOLOX, integrating a small target detection layer to enhance precision. Jajja et al. (2022) proposed a Compact Convolutional Transformer (CCT)-based approach is to classify the image dataset, achieving an impressive accuracy of 97.2% and proving its effectiveness compared to state-of-the-art approaches. Additionally, Patil and Patil (2021) developed a deep CNN model that accurately collected images throughout the complete process of training and validation in image pre-processing, ensuring high efficiency and accuracy for cotton disease detection. Liang, X (Liang, 2021) proposed a metric learning method for extraction and classification of cotton leaf spot characteristics. By constructing a metric space and using KNN as a point classifier, common models such as Vgg, DenseNet and ResNet were compared. The spatial structure optimizer (SSO) is introduced to perform local optimization of the model. Experimental results show that the average classification accuracy of S-DenseNet is 7.7% higher than the other two networks, and DenseNet shows the highest classification accuracy. Tao, Y et al (Tao et al., 2022). proposed an automatic detection method for cotton diseases, using ConvNeXt to combine the convolutional neural network architecture with the inherent advantages of Transformer. The Multi-Scale Spatial Pyramid Attention (MSPA) module can help ConvNeXt focus on important areas of feature maps. The results show that the model performs well in terms of recognition accuracy and detection speed.

In the realm of identifying pests, diseases, and behaviors using YOLO algorithms, extensive research has been conducted, highlighting their current significance. However, when applied to cotton pests and diseases identification, conventional YOLO algorithms encounter challenges in detecting cotton leaf diseases under natural conditions, difficulty in extracting features from small targets, and low efficiency (Terven and Cordova-Esparza, 2023). To overcome these challenges, this study presents an enhanced method for cotton peat and disease identification, built upon YOLOv8s (Xie and Sun, 2023). This method involves replacing the C2F modules in the backbone network with CFNet modules (Zhang G. et al., 2023) and substituting all C2F modules in the YOLOv8s header with VoV-GCSP modules (Li et al., 2022). It also integrates the LSKNet attention mechanism (Li et al., 2023) into the small target layers of both the backbone network and header. Furthermore, the XIoU loss function is introduced to streamline the model while preserving accuracy, ultimately enhancing the model’s convergence performance.

## 2 Materials and methods

### 2.1 Experimental data

**Figure 1:**
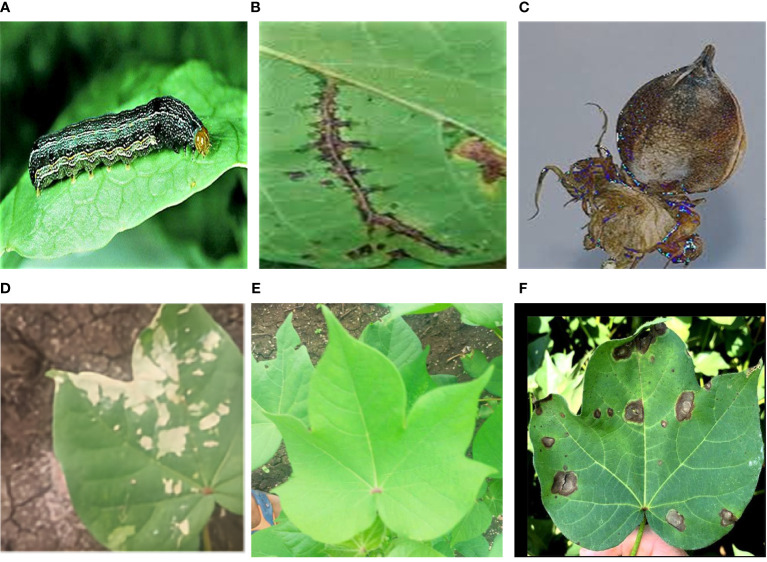
Examples of Images of **(A)** Nocturnal moth larvae **(B)** Cotton angular leaf spot **(C)** cotton boll rot **(D)** Cotton hoarfrost **(E)** Health and **(F)** Alternaria leaf spot of cotton.

The data used in this study were sourced from six publicly available cotton pest and disease datasets on KAGGLE (https://www.kaggle.com/datasets/saeedazfar/customized-cotton-disease-dataset; https://www.kaggle.com/datasets/paridhijain02122001/cotton-crop-disease-detection). Images that were blurry or had indistinct features were removed during data cleaning, resulting in a total of 4,703 images for pests and diseases, as illustrated in **Figure 1**. Due to data imbalance, data augmentation techniques such as rotation, brightness adjustment, and random cropping were applied (Tang et al., 2020), expanding the dataset to 5,927 images. The training and test datasets were then divided in an 8:2 ratio using random sampling, as shown in **Table 1** below. During training, the image size was set to 640×640 pixels.

**Table 1:** Information on cotton pest and disease data sets.

Pest and disease categories	Original data quantity	Quantity after expansion	Label
Army worm	799	799	Army_worm
Bacterial Blight	1136	1136	Bacterial_Blight_edited
Cotton Boll Rot	916	916	Cotton_Boll_Rot
Diseased cotton leaf	340	1020	diseased_cotton_leaf
Healthy	968	968	Healthy
Target spot	544	1088	Target_spot

### 2.2 Cotton pest and disease identification

#### 2.2.1 YOLOv8 network structure

**Figure 2:**
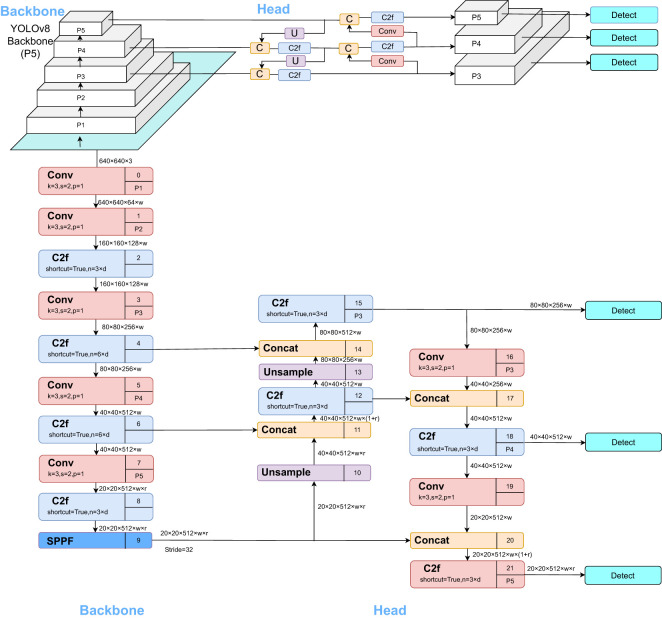
YOLOv8s Network Architecture.

YOLOv8 is a state-of-the-art (SOTA) model that builds upon the successes of previous YOLO versions, incorporating novel features and enhancements to further improve performance and versatility. Specific innovations include a new backbone network, a new Anchor-Free detection head, and a novel loss function. Anchor-Free Detection Head: Traditional object detection models utilize anchor boxes to determine the position and size of targets. In contrast, the Anchor-Free detection head learns the key-points or bounding boxes of the targets, thus eliminating the need for anchor boxes. This approach enables the model to better adapt to targets of varying sizes and shapes while reducing the complexity associated with tuning anchor boxes. Novel Loss Function: The loss function serves as feedback during training, assisting the model in fine-tuning its parameters for better target approximation. Currently, the YOLOv8 series has introduced five different versions, namely YOLOv8n, YOLOv8s, YOLOv8m, YOLOv8l, and YOLOv8x. The model’s parameter and computational complexity increase with the depth and width of the model. Users can choose the appropriate network structure based on their application scenarios. The YOLOv8s version employs a lighter network structure and fewer training data, aiming to maintain relatively fast detection speed and high accuracy while efficiently deploying on embedded devices and small applications (Wang G. et al., 2023). This makes YOLOv8s an ideal choice for real-time object detection applications. Therefore, this paper adopts the YOLOv8s model to meet the demand for efficient object detection. The YOLOv8 model detection network structure, as illustrated in **Figure 2** below, comprises the Backbone, FPN, and Head.

The Backbone serves as YOLOv8’s primary feature extraction network. Images fed into this network initially undergo feature extraction to produce what is commonly referred to as feature layers, a comprehensive set of features derived from the input images. These Feature Pyramid Network (FPN) in YOLOv8 is an augmented feature extraction component. Three significant feature layers obtained from the backbone network are further integrated in this section. The objective of this feature fusion is to combine feature information from various scales. The FPN continues to extract features from the already obtained significant feature layers. YOLOv8 still employs the Panet architecture, which not only up-samples the features for fusion but also down-samples them for an additional fusion. The Head in YOLOv8 serves as the classifier and regressor. Through the Backbone and FPN, we can obtain three enhanced, significant feature layers.

#### 2.2.2 CFNet-VoV-GCSP-LSKNet-YOLOv8s network structure

**Figure 3:**
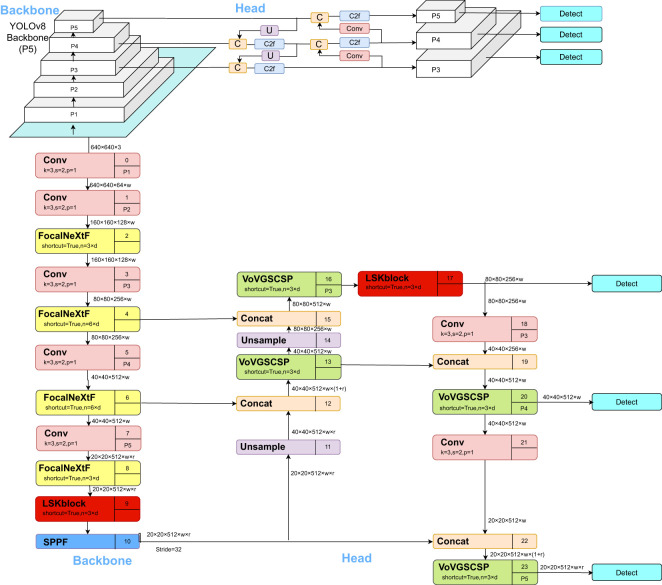
CFNet -VoV-GCSP-LSKNet-YOLOv8s network structure.

The network structure of the cotton pest and disease identification model based on CFNet-VoV-GCSP-LSKNet-YOLOv8s proposed in this paper is illustrated in **Figure 3**.

YOLOv8 is a state-of-the-art (SOTA) model, further categorized into YOLOv8n, YOLOv8s, YOLOv8m, YOLOv8l, and YOLOv8x. The YOLOv8 detection network structure consists of Backbone, FPN, and Head. While the Backbone has a large number of parameters and a long training time, it falls short in the detection of small objects. To address these limitations, we propose a cotton pest and disease identification model based on CFNet-VoV-GCSP-LSKNet-YOLOv8s. Firstly, the CFNet module replaces all C2F modules in the YOLOv8s Backbone. Then, a feature integration operation is inserted in the Backbone, effectively utilizing a large proportion of the Backbone to fuse multi-scale features, thereby improving the model’s recognition rate. Secondly, in the Head of YOLOv8s, the VoV-GCSP module replaces all C2F modules, enhancing the features extracted by the Backbone while also reducing the model size without sacrificing accuracy. Additionally, the LSKNet attention mechanism is incorporated into both the Backbone and Head to improve the detection of small objects. Lastly, the XIoU loss function is introduced to enhance model convergence, thereby achieving accurate identification of cotton pests and diseases.

#### 2.2.3 Cascaded fusion network

In the YOLOv8 Backbone, the C2F module attempts to fuse shallow feature maps with high resolution but limited semantic information with deep feature maps that have low resolution but rich semantic content. However, we argue that this approach might be insufficient for effective multi-scale feature fusion, especially when compared to heavy classification backbones where the parameters allocated for feature fusion are limited. To address this issue, we propose a new architecture named Cascaded Fusion Network (CFNet). Apart from the initial high-resolution feature-extracting Backbone and several blocks, we introduce multiple cascading stages to generate multi-scale features within CFNet. Each stage consists of a sub-backbone for feature extraction and an extremely lightweight transformation block for feature integration. This design allows for a more in-depth and effective fusion of features, leveraging a large proportion of the Backbone’s parameters. By replacing all C2F modules in the Backbone with CFNet and then inserting feature integration operations, we achieve effective fusion of multi-scale features across a significant portion of the Backbone.

The core design philosophy of CFNet involves introducing multiple cascading stages, each stage consisting of a specialized feature extraction sub-backbone and an extremely lightweight transformation block, effectively capturing and merging multi-scale features from fine-grained to coarse-grained. These cascading stages not only process the output from the previous stage but also deeply interact with the corresponding features of the main backbone network, achieving complex feature integration. The feature maps produced by each cascading stage are optimized and merged through specifically designed transformation blocks, enhancing the model’s ability to represent features. This structure is especially suitable for object detection tasks that require efficient multi-scale feature fusion. By optimizing and deeply integrating features at different levels, CFNet improves model performance while maintaining relatively low computational costs.

Suppose $X_{i}$ represents the output feature map of the ith cascading stage of the input image, where i represents the sequence number of the cascading stage. F represents the feature extraction function, and T represents the feature transformation function (lightweight transformation block). Thus, each cascading stage can be formally represented as shown in **Equation 1**:


(1) $$X_{i+1}=T\left(F\left(X_{\text{i}}\right)\right),\text{ for i}=0,1,\ldots ,M-1$$

In CFNet, M represents the total number of cascading stages. $X_{0}$ is the initial high-resolution feature map (as shown in **Equation 2**). For feature fusion, suppose $P_{j}$ denotes the j fused feature map, corresponding to different spatial resolutions, such as P3,P4,P5 etc. Each $P_{j}$ can be calculated through the output of the cascade and the corresponding transformation function $T_{j}$ .


(2) $$P_{j}=T_{j}\left(X_{M}\right),for j=3,4,5$$

**Figure 4:**
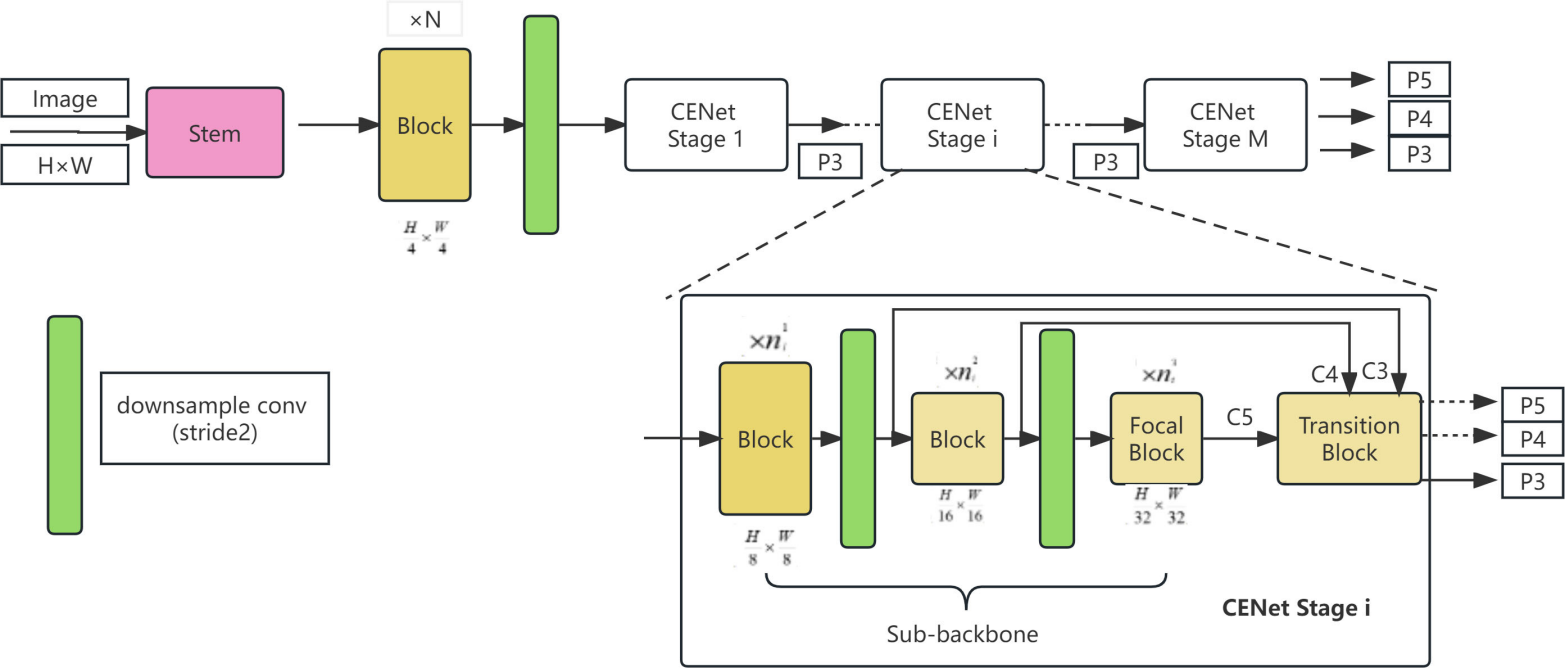
CFNet network architecture.

In CFNet,. refers to the output of the final cascading stage. The network architecture of CFNet is illustrated in **Figure 4**.

**Figure 5:**
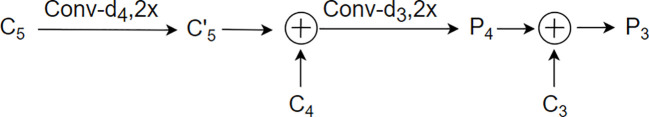
Transition block.

**Figure 6:**
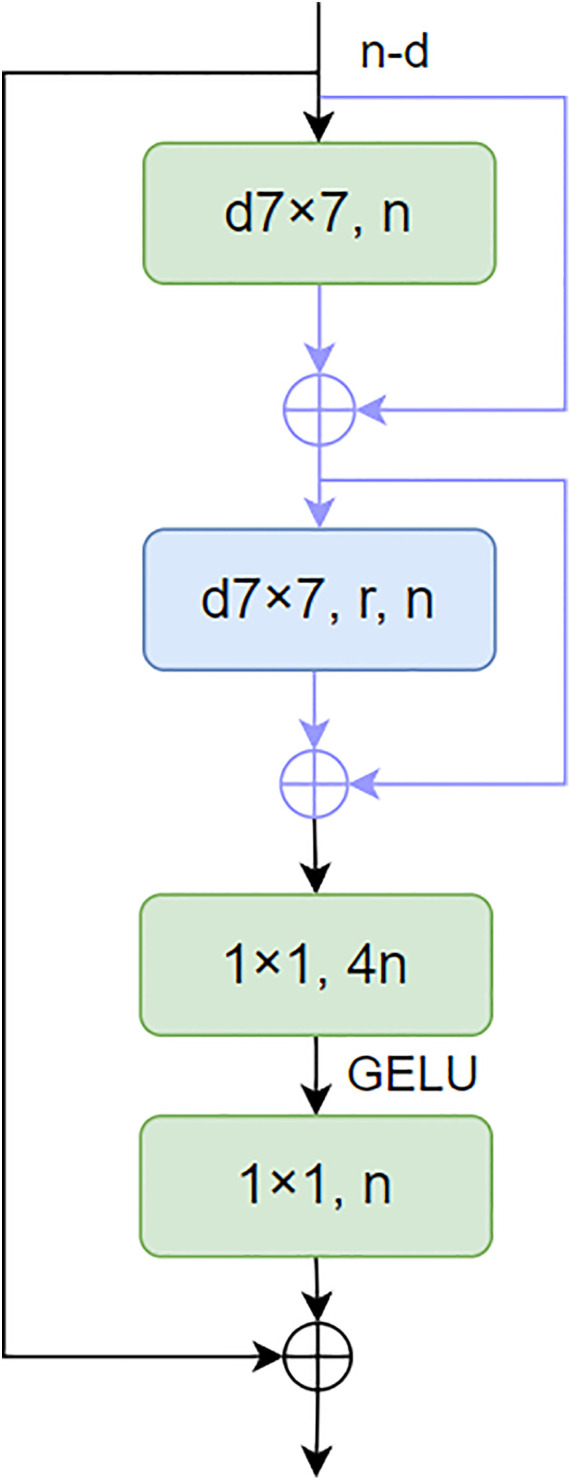
Focal NeXt block.

The CFNet architecture commences by inputting an image with spatial dimensions of H × W through a neck and N successive blocks, extracting high-resolution features with dimensions of $\frac{H}{4}\times \frac{W}{4}$ . These features are subsequently directed into M cascaded stages for the extraction of multi-scale features. Prior to entry into the M cascaded stages, the extracted high-resolution features are downscaled using a 2×2 convolution kernel with a stride of 2. The network’s architecture at each stage maintains a consistent structural format but varies in scale, comprising differing numbers of processing blocks. Each stage consists of a sub-backbone network and an ultra-lightweight transition block, both dedicated to the extraction and integration of features. For clarity, the assemblage of blocks within each stage, addressing features of the same scale, is termed a block group. The three block groups within the ith stage encompass ${\text{n}}_{i}^{1}$ , ${\text{n}}_{i}^{2}$ , and ${\text{n}}_{i}^{3}$ blocks, respectively. In the final block group of each stage, a so-called focal block is implemented to enhance feature processing. Each stage outputs features P3, P4, P5 with strides of 8, 16, 32, respectively, of which only P3 features are utilized for input into the subsequent stage. In the network’s final stage, features P3, P4, and P5 are amalgamated, serving dense prediction tasks. By substituting all C2F modules in the backbone with CFNet and incorporating feature integration operations, effective fusion of multi-scale features is achieved throughout a significant portion of the backbone. **Figures 5** and **6** provide more details about transition blocks and focus blocks.

As depicted in **Figure 5**, When given C_3_, C_4_, and C_5_ as inputs, the transition block produces outputs P_3_ and P_4_. The term “Conv -dx” refers to a 1×1 convolution operation that outputs a channel number of dx, where dx matches the channel number of the input feature C_x_. The circles marked with “+” and “C” represent element-wise addition and concatenation operations, respectively. Additionally, the notation “2x” is used to indicate the upsampling of features by a scaling factor of 2.

This design facilitates the effective integration of multi-scale features. By adjusting the channel dimensions through 1×1 convolutions and managing the spatial resolutions via addition, concatenation, and upsampling operations, the transition block efficiently processes the varied scales of the input features (C_3_, C_4_, C_5_) and transforms them into the desired output formats (P_3_, P_4_), which are then suitable for subsequent stages of the network’s processing pipeline.

As shown in **Figure 6**, N is the number of channels of the output feature. d7×7 represents the 7×7 depth convolution, a7×7 represents the window size, and R is the expansion rate of the additional convolution. GELU is the activation function. Each d7×7 or a7×7 is followed by a LayerNorm layer and a GELU unit. This paper proposes a novel focus block to enlarge the receptive fields of neurons in the last block group of each stage as an effective alternative strategy. The design of the focus module introduces extended depth convolution and two skip connections in the ConvNeXt module, thereby achieving the integration of fine-grained local interaction and coarse-grained global interaction.

#### 2.2.4 VoV-GCSP network structure

The YOLOv8 network employs a substantial number of C2F modules in its neck for feature extraction. However, this structure results in an increase in computational complexity and the number of parameters, leading to significant time consumption. Lightweight networks like Exception and ShuffleNet address the time-consuming issue of standard convolutions by utilizing depth-wise separable convolutions (DSC), albeit at the cost of sacrificing accuracy. The GSConv convolution module is an innovative approach that combines Standard Convolution (SC), Depth-Wise Convolution (DWConv), and channel shuffle operations. The core idea involves partitioning the input channels into multiple groups, performing independent depth-wise separable convolutions on each group to reduce computational complexity. This design aims to mitigate the issue of low recognition accuracy due to insufficient feature extraction and fusion capabilities. The groups are then recombined through channel shuffling. GSConv combines SC, DSC, and Shuffle, exhibiting performance similar to SC but with lower computational costs. The depth layer calculation is shown in **Equation 3**, and the GSConv layer calculation is shown in **Equation 4**.


(3) $$\text{DSC}=\text{W}\times \text{H}\times \text{m}\times \text{n}\times 1\times {\text{P}}_{\text{out}}$$

(4) $$\text{GSConv}=\text{W}\times \text{H}\times \text{m}\times \text{n}\times 1\times \frac{{\text{P}}_{\text{out}}}{2}+({\text{P}}_{\text{out}}+1)$$

**Figure 7:**
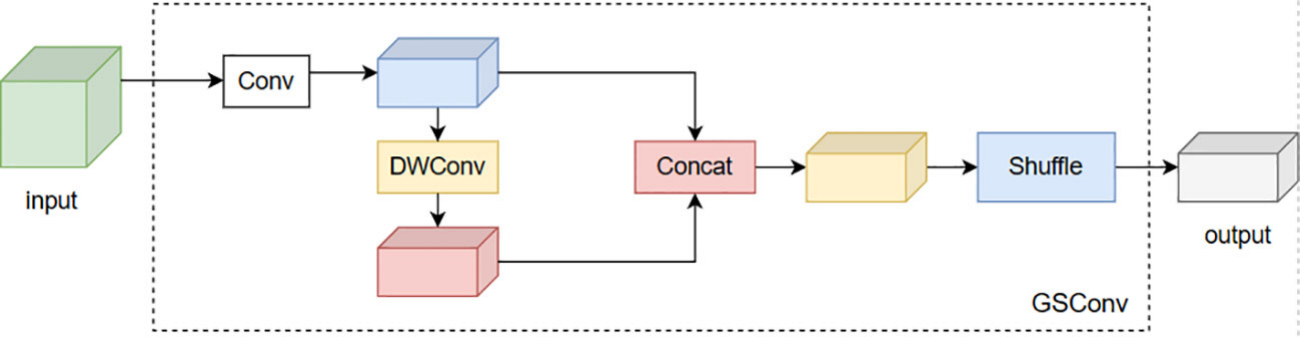
GSConv.

**Figure 8:**
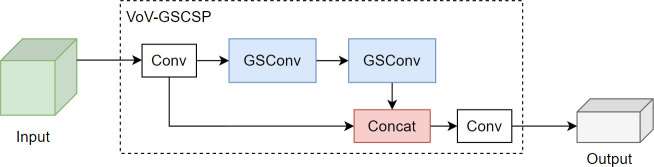
VoV-GSCSP.

W and H represent the width and height of the feature map, respectively, and m×n is the size of the convolution kernel. Pin and Pout represent input and output function channel numbers. In scenarios where the input feature channel count escalates, the computational demand of the GSConv convolution diminishes, yet it retains a feature extraction proficiency analogous to its contemporaries. The integration of GSConv has been instrumental in the strategic simplification of the model’s complexity. To augment the inference velocity of the network model, while concurrently preserving its precision in detection, we have implemented the VoV-GSCSP module, building upon the foundational GSConv module. The VoV-GSCSP represents a sophisticated hybrid network architecture, which skillfully merges the attributes of GSConv with the essence of VoVNet, supplemented by the incorporation of (Squeeze-and-Excitation, SE) blocks. This architectural design is meticulously tailored to enhance both the quality and efficiency of feature extraction. By segmenting the convolutional layers into discrete groups, the Grouped Separable Convolution effectively minimizes the parameter count and computational complexity. The Squeeze-and-Excitation blocks intensify the network’s representational prowess by concentrating on salient channel features. This innovative structural design endows the VoV-GSCSP module with the capability to sustain high computational efficiency while simultaneously elevating the feature representation and overall performance of the network. The module, engineered with a one-off aggregation methodology, markedly amplifies the inference speed of the network model, all the while maintaining its superior detection accuracy. The configurations of the GSConv convolution and the VoV-GCSP network are exemplified in **Figure 7** and **Figure 8**.

#### 2.2.5 Attention mechanism LSK

**Figure 9:**
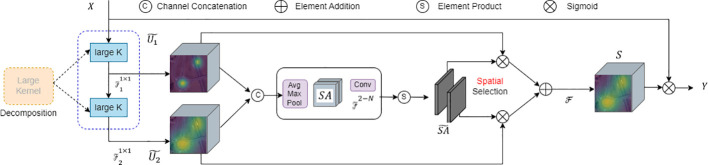
Conceptual diagram of the LSK module.

Attention mechanisms serve as a straight forward effective approach yet to enhance neural representations. Channel attention modules like SE blocks utilize global average information to re-weight feature channels, while spatial attention modules such as GENet, GCNet, and SGE enhance the network’s capability to model contextual information via spatial masks. Techniques like CBAM and BAM amalgamate channel and spatial attentions, leveraging the strengths of both. Beyond channel/spatial attention mechanisms, kernel selection is another adaptive and effective technique for dynamic contextual modeling. LSKNet is designed based on attention mechanisms and kernel selection technologies to better model the features of different targets in remote sensing scenarios. It also boasts advantages like relatively fewer parameters and computational complexity, thereby facilitating improved computational efficiency and speed in practical applications. LSKNet is a novel neural network architecture specifically aimed at remote sensing object detection tasks. It enhances contextual modeling and feature extraction through selective mechanisms and adaptive spatial aggregation, consequently improving the performance in small object detection. A detailed structural comparison is shown in **Figure 9**.

#### 2.2.6 XIoU

The Loss Function is a metric that measures the difference between the predicted values of a model and the actual values. During training, the model attempts to minimize the value of the loss function to improve its accuracy. YOLOv8s adopts the CIoU loss function, composed of position, confidence, and class functions. This traditional loss function generally relies on the aggregation of bounding box regression indicators, without considering the mismatch in direction between the required ground truth boxes and predicted boxes, leading to slow convergence and low efficiency. The XIoU loss function plays a crucial role in object detection tasks by emphasizing varying degrees of overlap between targets. By combining the regression of predicted boxes with real boxes, this loss function prevents issues such as overlapping center points and identical aspect ratios that would degrade into the IOU loss function. This ensures the effective completion of boundary box regression, improving the robustness of the bounding boxes. Therefore, in this study, the XIoU loss function is introduced as an improvement to the model. Compared to the original CIoU loss function, the penalty term gradient of XIoU is smoother, resulting in smaller regression errors and better regression performance. It also effectively enhances the recognition accuracy of cotton leaf diseases and pests.

XIoU calculation formula is as shown in **Equation 5**:


(5) $$XIoU=1-IoU+\frac{\rho^{2}\left(b,b^{gt}\right)}{c^{2}}+\alpha \upsilon$$

(6) $$\alpha =\frac{\nu }{\left(1-IoU\right)+\nu }$$

(7) $$\nu ={\left(e^{\frac{w^{gt}}{h^{gt}}}-e^{\frac{w}{h}}\right)}^{2}$$

The penalty term is defined as shown in **Equation 8**:


(8) $${\text{ℜ}}_{CIoU}=\frac{\rho^{2}\left(b,b^{gt}\right)}{c^{2}}+\alpha \upsilon$$

As shown in **Equations 5**–**8**, IoU stands for the traditional regression loss. $\rho^{2}$ represents the squared Euclidean distance between the two rectangular bounding boxes. $c^{2}$ represents the square of the diagonal distance between two rectangular boxes. b and *b^gt^* denote the central points of the two bounding boxes. α weight coefficient.*v* is used to measure the consistency of the relative proportions between the two boxes. *w^gt^*, *h^gt^*, *w* and *h* are the width and height of the two boxes, respectively. The primary goal of XIoU is to improve the IoU metric by considering the intersection area between the boxes, offering a better representation of their overlap. The parameter *α* is used to adjust the difference between XIoU and IoU, thereby reflecting the similarity between the boxes more accurately and accelerating the network’s convergence.

## 3 Results and discussion

### 3.1 Improve model identification results and analysis

#### 3.1.1 Experimental setup and evaluation metrics

The model was trained using the PyTorch framework on a laboratory server equipped with an Intel Core i9-10900KF processor, 16 GB of CPU memory, and an NVIDIA GeForce RTX 3080 GPU. The operating environment was Windows 10, with Python 3.8, PyTorch 1.11.0, and CUDA 13.0 used for algorithmic optimization. Training parameters included 150 epochs, a batch size of 8, and an image input resolution of 640×640 pixels. All other settings were kept at their default values.

Performance metrics used for model evaluation included Precision (P), Recall (R), Mean Average Precision (mAP), and model size. Precision is defined as the fraction of true positives among the predicted positives, while Recall measures the fraction of actual positives correctly identified by the model. Mean Average Precision (mAP) serves as a comprehensive performance metric. The above indicators such as **Equations 9**–**12** shown.


(9) $$P=\frac{TP}{TP+FP}$$

(10) $$R=\frac{TP}{TP+FN}$$

(11) $$AP=\int_{0}^{1}P(r)\text{dr}$$

(12) $$mAP\;=\;\frac{1}{n}\sum_{i-1}^{\text{n}}AP_{i}$$

In the equations, TP represents the number of true positives, FP stands for false positives, and FN signifies false negatives.

#### 3.1.2 Cotton pest and disease recognition results

To validate the superior performance of the proposed CFNet- VoV-GCSP -LSKNet -YOLOv8 architecture for the identification of six types of cotton pests and diseases, we compared our model with the original YOLOv8s algorithm, as shown in **Table 2**.

**Table 2:** Comparison of average precision mean values for cotton pests and diseases.

Cotton pest and disease categories	mAP_@0.5/%_	
	Proposed Method	YOLOv8s
Nocturnal moth larvae	98.6	97.7%
Cotton angular leaf spot	97.1	96.5
cotton boll rot	99.5	99.4
Cotton hoarfrost	98.5	96.1
Health	92.0	90.9
alternaria leaf spot of cotton	76.5	74.3
All pests and diseases	93.7	92.5

From **Table 2**, it can be observed that the method proposed in this paper for identifying six types of cotton pests and diseases—namely, noctuid larvae, cotton angular leaf spot, cotton boll rot, cotton powdery mildew, healthy cotton, and cotton black spot—achieves an average precision mean (mAP@0.5) improvement compared to the original model of 0.9%, 0.6%, 0.1%, 2.4%, 1.1%, and 2.2%, respectively.

**Figure 10:**
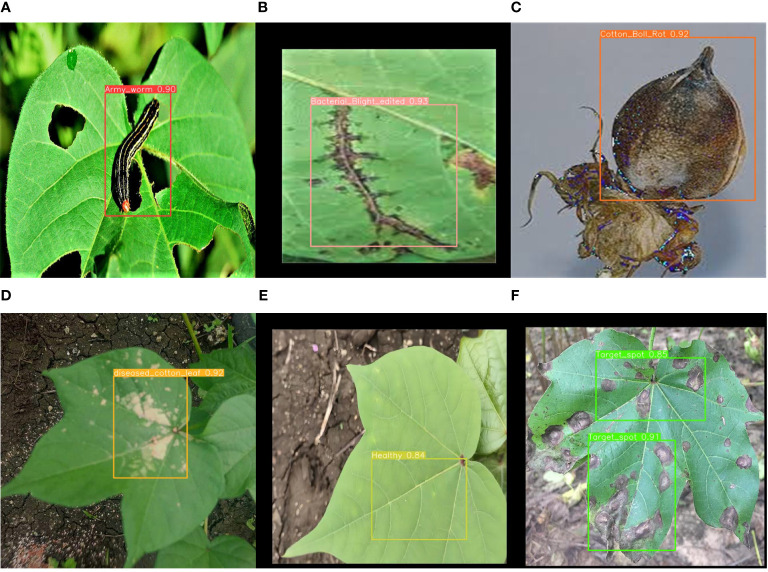
Recognition results of this paper’s method: **(A)** Nocturnal moth larvae **(B)** Cotton angular leaf spot **(C)** cotton boll rot **(D)** Cotton hoarfrost **(E)** Health and **(F)** Alternaria leaf spot of cotton.

Among the six types of cotton pests and diseases, the average precision mean (mAP@0.5) for cotton black spot is the lowest, with only 74.3%. Analysis indicates that the blurriness of the original data images led to this subpar performance. However, with the application of our method, there is a 2.2% improvement over the original model, resulting in an overall average precision mean (mAP@0.5) of 93.7%, an increase of 1.2% compared to YOLOv8s. This demonstrates that our model’s feature extraction capability has been enhanced for images with suboptimal quality. The results of the method proposed in this paper for identifying cotton pests and diseases are shown in **Figure 10**:

#### 3.1.3 Ablation study results

To validate the efficacy of the improvements made to the original algorithm by the cotton pest and disease identification method based on the CFNet-VoV-GCSP-LSKNet-YOLOv8s network structure proposed in this paper, an ablation study was designed. The original network and the network improved with various modules were tested on a test dataset. The results are shown in **Table 3**:

**Table 3:** Ablation test results.

Model	Baseline model	CFNet	VoV-GCSP	LSKNet	XIoU	P/%	R/%	mAP_@0.5/%_
Model 1	YOLO v8s	**×**	**×**	**×**	**×**	87.9	89.7	92.5
Model 2	YOLO v8s	**√**	**×**	**×**	**×**	90.6	89.4	93.3
Model 3	YOLO v8s	**×**	**√**	**×**	**×**	87.4	90.7	92.9
Model 4	YOLO v8s	**×**	**×**	**√**	**×**	88.9	88.2	92.7
Model 5	YOLO v8s	**√**	**√**	**×**	**×**	89.1	89.8	93.4
Model 6	YOLO v8s	√	√	√	√	89.9	90.7	93.7

1. Model 2, the first improvement, replaces all C2F modules in the backbone with CFNet modules, resulting in a 2.7% increase in Precision (P), a 0.3% increase in Recall (R), and a 0.8% increase in Mean Average Precision (mAP). This indicates that the CFNet module effectively fuses multi-scale features and improves model recognition accuracy.
2. Model 3 replaces all C2F modules in the YOLOv8s head with VoV-GCSP modules, leading to a 1% increase in R and a 0.4% increase in mAP. However, P decreased by 0.5%, but the overall performance is still better than the original model, suggesting that the neck structure composed of VoV-GCSP modules enhances the features extracted by the backbone.
3. Model 4 adds the LSKNet attention mechanism compared to YOLOv8s, resulting in a 1% increase in P and a 0.2% increase in mAP, suggesting that the LSKNet attention mechanism strengthens the model’s ability to recognize small objects.
4. Model 5 incorporates both CFNet and VoV-GCSP modules, leading to a 1.2% increase in P, a 0.1% increase in R, and a 0.9% increase in mAP.
5. Model 6, based on the improvements in Model 5, further incorporates the LSKNet attention mechanism and replaces the loss function with XIoU. It turns out that Model 6 has the highest precision among all the models. Compared to the original model, it increases P by 2%, R by 1%, and mAP by 1.2%, demonstrating that the improved model outperforms YOLOv8s in recognition performance and effectively enhances cherry detection capabilities.

**Figure 11:**
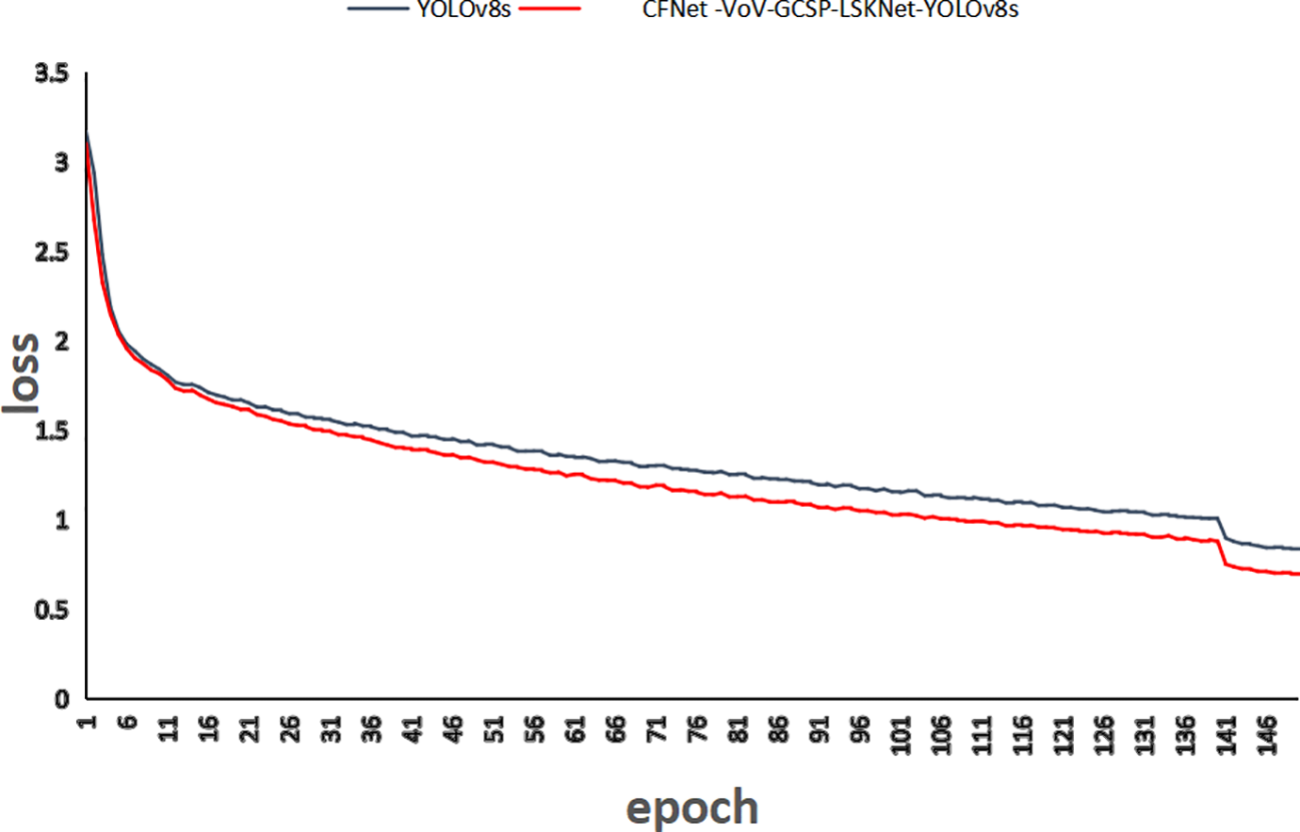
Comparison of positional loss values.

To further validate the effectiveness and practicality of the method proposed in this paper, the location loss values of CFNet-VoV-GCSP-LSKNet-YOLOv8s and YOLOv8s are shown in **Figure 11** after 150 training iterations. As can be seen from **Figure 11**, the convergence speed of the proposed method is faster, and its convergence performance is superior to that of the YOLOv8s model.

#### 3.1.4 Comparison of different models

To verify the effectiveness of the cotton pest and disease identification method based on the CFNet-VoV-GCSP-LSKNet-YOLOv8s model proposed in this paper, we compared it with YOLO v5s (Jiang et al., 2022), YOLOX (Ge et al., 2021; Zhang et al., 2022b), YOLOv7 (Cao et al., 2023; Wang C-Y. et al., 2023), Faster R-CNN (Li, 2021; Qiao et al., 2021).

YOLO v8s, YOLOv8n, YOLOv7-tiny, CenterNet and EfficientDet. Among them, YOLOv8s, YOLO v5s, YOLOv7, YOLOv8n, YOLOv7-tiny, CenterNet and EfficientDet are currently mainstream object detection algorithms, while YOLO X and Faster R-CNN have shown better performance in other studies. To validate the superiority of the proposed method, all model training processes maintained consistent parameter settings. The comparison results are shown in **Table 4**.

**Table 4:** Comparison of recognition effect of different models.

Model	P/%	R/%	mAP_@0.5/%_	volume modulus/MB	Detection Time/ms
YOLO v5s	88.5	88.8	91.4	14.4	10
YOLOX	90.6	86.7	88.7	34.4	9.1
YOLO v7	78.5	79.1	81.2	74.8	11.2
Faster R-CNN	60.8	91.2	84.7	521.9	9.9
YOLO v8n	84.4	85.3	90.0	6.2	7.0
YOLOv7-tiny	87.7	41.5	71.9	23.5	8.2
CenterNet	95.8	65.1	88.8	124	10.4
EfficientDet	87.8	70.7	81.6	25.7	9.3
YOLO v8s	87.9	89.7	92.5	21.4	7.7
Proposed Method	>89.9	>90.7	>93.7	>23.3	8.01

**Figure 12:**
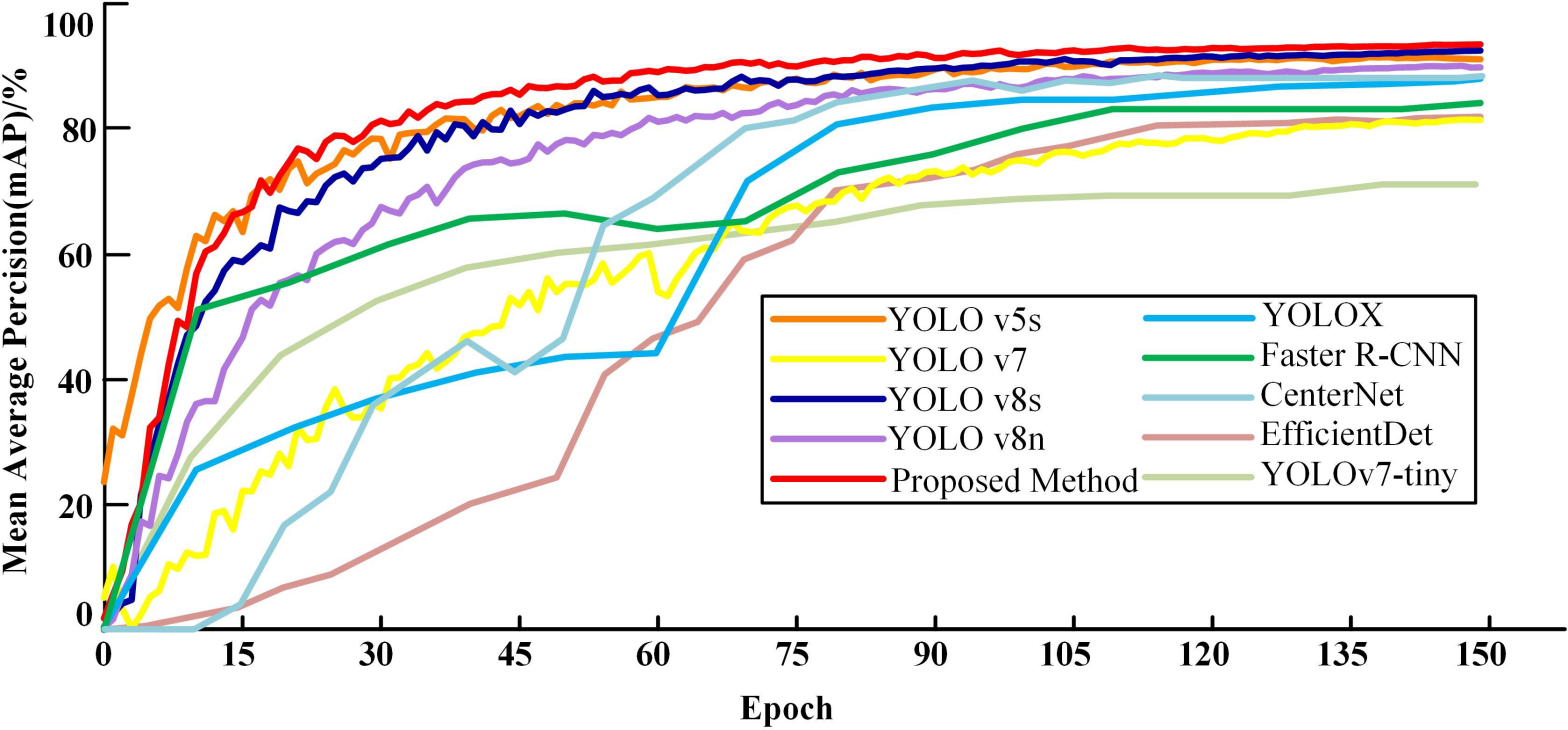
Variation curves of mAP for different models.

As delineated in **Table 4**, the proposed method demonstrates marked improvements in performance metrics over existing methods. Specifically: When compared with YOLO v5s, YOLO v7, YOLOv8n, YOLOv7-tiny, EfficientDet and YOLOv8s, the precision of our model improved by 1.4%, 11.4%, 5.5%, 2.2%, 2.1% and 2% respectively. Additionally, recall rates saw increases of 1.9%, 11.6%, 5.4%, 49.2%, 20% and 1%, and the mean average precision (mAP) advanced by 2.3%, 12.5%, 3.7%, 21.8%, 12.1% and 1.2%. While our method registers a slight decline in precision relative to YOLOX, it compensates with a 4% increase in recall and a 5% boost in mAP. As for Faster R-CNN, although the recall rate was marginally lower by 0.5%, the model achieved a substantial enhancement in precision by 29.1%, along with a 9% improvement in mAP. Compared with CenterNet, although the precision is 5.9% lower, the mAP is 4.9% higher and the recall rate is 25.6% higher. In terms of computational resource consumption, our model is second only to YOLO v5s, YOLOv8n and YOLO v8s. The detection time of the improved algorithm is 8.01ms. Although slightly lower than the fastest detection speed YOLOv8n and YOLO v8s, other performance indicators of the detection algorithm are better than this model. Therefore, based on the overall detection performance indicators of the model, the algorithm in this paper has great advantages in both recognition accuracy and speed. Collectively, these results validate the effectiveness of the proposed method, positioning it as superior in object detection performance. **Figure 12** offers further insights into the comparative performance of various models. While the proposed method slightly lags behind YOLO v5s in terms of convergence speed during the initial 17 iterations, it surpasses all competing models in both convergence speed and mAP following the 17th iteration.

## 4 Discussion

In order to verify the robustness and effectiveness of the model, the YOLOv8s original model and the Proposed Method were tested on datasets collected from the Kaggle website, which include grape and coffee disease datasets. The grape disease dataset consists of four types of diseases (Black Rot, Grape Esca, Grape Healthy, and Leaf Blight), totaling 3330 images. The coffee disease dataset consists of ten types (Coffee White Stem Borer, Citrus Mealybug, Coffee Berry Borer, Coffee Root-knot Nematode, Coffee Berry Moth, Coffee Leaf Miner, Coffee Twig Borer, Coffee Seedling Sudden Collapse Disease, Coffee Seedling Damping-off Disease), totaling 4500 images. The experimental results are shown in the table below.

As shown in the table above, the precision, recall rate, and mAP of Proposed Method have improved in grape disease identification, increasing by 0.1%, 0.1%, and 0.15% respectively. The precision, recall rate, and mAP of Proposed Method in identifying coffee diseases have been greatly improved, increasing by 1%, 2.8%, and 1.8% respectively. It shows that the Proposed Method has a good recognition effect on public plant disease data sets, thus proving that the model has good robustness and effectiveness. In the future, the Proposed Method can be applied in various fields.

## 5 Conclusions

This paper proposes a cotton pest and disease recognition method based on CFNet-VoV-GCSP-LSKNet-YOLOv8s. By replacing all C2F modules in the backbone of YOLO v8s with CFNet modules and incorporating feature fusion operations, the method effectively utilizes a significant proportion of the backbone network to fuse multi-scale features. In the head of YOLOv8s, we replaced all C2F modules with VoV-GCSP modules. This enhances the features extracted by the backbone while reducing the model’s complexity and maintaining its accuracy. We also introduced the LSKNet attention mechanism in both the backbone and the head to improve the model’s ability to recognize small targets. Finally, the XIoU loss function was introduced to improve the model’s convergence performance. As shown in **Table 5**: experimental results show that the proposed method can effectively identify cotton pests and diseases with an average accuracy of 93.7%, demonstrating its effectiveness.

**Table 5:** Comparison of results for different diseases.

Data	Model	P/%	R/%	mAP_@0.5%_
Grape diseases	YOLOv8s	99.30	99.60	99.25
	Proposed Method	99.40	99.70	99.40
Coffee diseases	YOLOv8s	80.20	76.30	81.80
	Proposed Method	81.20	79.10	83.60

Compared with YOLO v5s, YOLOX, YOLO v7, Faster R-CNN, YOLOv8n, YOLOv7-tiny, CenterNet, EfficientDet and YOLOv8s, the average accuracy improved by 2.5%, 5%, 12.5%, 9%, 3.7%, 21.8%, 4.9%, 12.1% and 1.2% respectively, indicating that the method proposed in this paper performs better in recognizing cotton pests and diseases. In addition, the model in this article was applied to grape and coffee diseases, which greatly improved the disease identification rate, indicating that the model has good robustness and effectiveness.

While CFNet-VoV-GCSP-LSKNet-YOLOv8s demonstrates considerable prowess in numerous domains, there is still potential for advancement in its efficacy in detecting cotton leaf spot disease. Looking forward, our ambition is to enrich YOLOv8s’s backbone network with a sophisticated multi-channel scale attention mechanism, aimed at enhancing the precision in capturing the characteristics of plant diseases. Concurrently, by refining the final prediction bounding box optimization and the Adam optimizer within YOLOv8s, we aspire to elevate the model’s recognition proficiency. Moreover, in tandem with real-world application demands, our objective includes the development of mobile applications. This endeavor is geared towards translating our research findings into pragmatic tools, offering robust and practical solutions in the realms of agriculture and plant protection, thereby facilitating the deployment of this technology in real-world scenarios.

## Data availability statement

Publicly available datasets were analyzed in this study. This data can be found here: https://www.kaggle.com/datasets/paridhijain02122001/cotton-crop-disease-detection and https://www.kaggle.com/datasets/saeedazfar/customized-cotton-disease-dataset.

## Author contributions

RL: Conceptualization, Methodology, Resources, Validation, Writing – original draft. YH: Data curation, Methodology, Software, Validation, Writing – original draft, Writing – review & editing. YL: Data curation, Resources, Validation, Writing – review & editing. WQ: Data curation, Software, Writing – review & editing. AA: Data curation, Writing – original draft, Writing – review & editing. RJ: Data curation, Methodology, Writing – review & editing. SL: Methodology, Writing – review & editing. YW: Data curation, Writing – review & editing. XS: Software, Writing – review & editing. JY: Supervision, Writing – review & editing.
